# High-content molecular profiling of T-cell therapy in oncology

**DOI:** 10.1038/mto.2016.9

**Published:** 2016-03-30

**Authors:** Ruslan Novosiadly, Michael Kalos

**Affiliations:** 1 Department of Cancer Immunobiology, Eli Lilly and Company, New York, New York, USA

## Abstract

Recent clinical data have revealed the remarkable potential for T-cell-modulating agents to induce potent and durable responses in a subset of cancer patients. In this review, we discuss molecular approaches, platforms, and strategies that enable a broader interrogation of the activity of agents that modulate the activity of tumor-specific T cells, to more comprehensively understand how and why the agents succeed and fail, as well as examples of data sets generated in clinical trials that have provided important insights into the biological activity of T-cell therapies and that support further rational development of this exciting treatment modality.

Immunotherapy strategies that modulate the T-cell response to cancer have emerged as attractive therapeutic modalities for the treatment of various human malignancies. Promising approaches have included: (i) augmenting and manipulating the ability of T cells to recognize tumors by the adoptive transfer of *ex-vivo* expanded T cells, either nonmodified or following genetic engineering to express chimeric antigen receptor (CAR) or T-cell receptor (TCR)^1,2^; (ii) the use of bispecific T-cell redirecting molecules such as bispecific T-cell engagers (BiTEs) and immune-mobilizing monoclonal TCRs against cancer (immTACs)^3,4^; and (iii) unleashing and enhancing existing endogenous antitumor T-cell responses through the targeting of immune checkpoint inhibitor and costimulatory agonist receptors agonists.^5,6^ Collectively, these approaches have demonstrated the potential of T-cell-based immunotherapy to significantly enhance clinical outcomes for cancer patients. Over the past few years, the remarkable clinical efficacy reported for T-cell-modulating strategies has led to multiple designations for breakthrough therapy, and accelerated approval timelines for a number of these agents across multiple tumor indications. Nevertheless, for each of these approaches, numerous outstanding issues still remain to be understood and addressed in order to capture their full potential to effectively treat disease.

Intuitively, the presence of therapy-relevant and effector-competent T cells at the tumor would seem to be a fundamental prerequisite for treatment efficacy of T-cell-based immunotherapies. Indeed, for both T-cell redirecting and T-cell-modulating strategies, the presence of relevant T cells has been positively associated with treatment efficacy.^7–9^ Beyond the essential issue of T-cell presence, the major challenges that have been identified as relevant for maximal efficacy of T-cell therapies include the need of long-term functional persistence of tumor-specific T cells, and understanding and mitigating the multitude of immunostimulatory and immunosuppressive mechanisms to modulate T-cell activity in the tumor microenvironment.^10^ In addition, the ability to interrogate the quality and breadth of immune modulation in response to treatment within and among patients offers the possibility to follow and address both treatment efficacy and potential toxicities in an effective manner.

The implementation of broad and systematic biomarker strategies is now recognized to be a key component to the successful development of immunotherapy agents.^11^ Molecular platforms, due to their inherent sensitivity, high-content and/or high-throughput potential, low-sample requirements, and relative ease for quality-enablement are ideally suited to support the broad and systematic interrogation of immunotherapy protocols to understand why, how, and when treatments succeed and fail.^12^ The development of new molecular platforms combined with technological advancements in existing platforms and assays have enabled the ability to comprehensively analyze a broad range of predictive, mechanistic, pharmacodynamic, and safety biomarkers during early clinical trials to enable successful development of T-cell-based therapies (Figure 1). In this review, we will focus on an overview and description of multiplex molecular and biochemical platforms that support the empiric development of T-cell-redirecting and -modulating strategies to effectively target cancer.

## T-Cell Redirection

T-cell recognition of tumor cells is an essential prerequisite for the success of T-cell immunotherapy strategies. To date, the majority of targeted tumor antigens have been differentiation or tissue-restricted self-antigens normally expressed during development and aberrantly expressed in tumor cells. It is now broadly recognized that T cells which recognize self-antigen-derived peptides typically express TCRs with low affinity for cognate major histocompatibility complex/peptide complexes as a consequence of central tolerance, resulting in a lack of the robust T-cell activation and poor antitumor activity, and a need for TCR affinity-enhancement for effective antitumor activity.^2^ More recently, the identification and clinical application of T cells specific for neoantigens, antigens which are derived from various nonsynonymous somatic mutations that occur spontaneously in cancer cells,^1^ has linked earlier and more recent associations between extent of T-cell infiltration, mutational burden, and response to immunotherapy,^13–15^ and has provided cause for considerable but guarded optimism that T cells with native receptors can mediate potent antitumor activity. Robust functionality of tumor-specific T-cell clones can additionally be blocked as a result of checkpoint-mediated immunosuppression, T-cell exhaustion, or by the immunosuppressive tumor microenvironement. Chronic exposure of engineered T cells to the antigen results in T-cell exhaustion and inability to proliferate, while recent reports demonstrate that immune checkpoints are expressed on CAR T cells after infusion.^16,17^

Several cellular and molecular engineering strategies have been pursued to overcome immune tolerance to tumor-specific self-antigens and redirect autologous T cells to effectively target antigen-positive tumor cells. Effective T-cell redirection can be enabled through synthetic-biology-based genetic engineering and transgenic expression in autologous T cells of antigen-specific αβTCR (potentially affinity enhanced), or CAR followed by adoptive T-cell transfer.^18,19^ An alternate strategy for T-cell redirection involves the development of recombinant proteins that bridge tumor cells to nonspecifically activated T cells. Well-studied and clinically validated antibody constructs for engaging T cells are BiTEs,^20^ which are based on single chain antibodies against specific antigens on tumor cells and effector molecules (*e.g.*, CD3) on T cells. BiTEs can transiently tether resting T cells to tumor cells, leading to concomitant T-cell activation and tumor cell lysis and showed promising antitumor activity in the clinic.^21^ ImmTACs represent another class of engineered proteins capable of redirecting T cells to the tumor.^22^ ImmTACs bi-specific molecules with picomolar affinity for TCRs fused to an anti-CD3 specific scFv. Although this class of T-cell redirecting agents triggers antitumor activity via nonspecific (*i.e.*, anti-CD3 based) engagement of T cells, durable antitumor activity is likely to depend on establishment of a secondary antigen-specific T-cell response, via epitope spreading or related immunological sequelae to the initial antitumor activity.

## Molecular Approaches to Track T Cells

As described above, both genetic and biochemical strategies for T-cell redirection likely require long-term persistence of functional tumor-specific T cells in the periphery and importantly also at sites of disease.

A number of techniques have been commonly used to detect and quantify antigen-specific T cells. Among these, flow cytometry, quantitative polymerase chain reaction (qPCR), Vβ spectratyping, high-throughput sequencing, and immunohistochemistry (IHC) have provided useful and relevant information about antitumor T-cell immunity. Flow cytometry is capable of analyzing cell surface expression at a single cell level but is analytically challenging, labor-intensive and has suboptimal sensitivity. Because of technical limitations of flow cytometry-based approach, detection of specific TCR α/β pairs present on infused cells typically has a quantification limit of 0.2–0.5% of the total CD3+ T-cell population. Given that CAR constructs contain a unique antibody fragment, idiotype-specific antibodies can be used to detect and quantify genetically modified T cells,^23^ and this approach was successfully implemented by multiple groups.^24–26^

qPCR-based platforms detect a unique molecular tag to identify the cells of interest. qPCR has much higher sensitivity and is capable of detecting genetically engineered cells at a very low frequency (~0.01% of total T cells). At present, it is a gold standard technique to evaluate persistence of engineered T cells *in vivo*.^24^ While qPCR is a highly sensitive and highly quantitative method, it does not provide any information about the phenotype and function of the persisting T cells. qPCR has been extensively used to track DNA sequences that are unique to adoptively transferred genetically modified T cells.^24,27–30^ T-cell products generated through mRNA electroporation require reverse transcription of RNA into DNA prior to qPCR analysis.^31^ Digital PCR (dPCR) is an alternative to qPCR that can be utilized if single cell analysis is required.

Both flow cytometry and qPCR can be employed to indirectly assess, with platform-associated sensitivity, the relative quantity of infused T cells using TCR spectratyping by evaluating the variable segment of the TCRβ (Vβ) usage.^32^ TCR spectratyping is predicated on the observation that the *TCRB* locus has multiple Vβ segments grouped into 25 Vβ families, with each Vβ family representing ~0.2–5% of the total T-cell population.^33^ The ability to effectively employ this approach depends on mono- or oligoclonal expansion of antigen-specific T cells, without a requirement for genetic modification, with detection of an antigen-specific T-cell repertoire based on the deviation from T cell “evenness”, as represented by a normal distribution of T cells from each Vβ family.

Antigen-specific T-cell populations can be successfully monitored by next-generation sequencing (NGS) and its modification, immunosequencing (immunoSEQ), an approach that allows for accurate quantification based on unique nucleotide sequences of genetically rearranged TCR.^30^ ImmunoSEQ is a multiplex PCR-based method that amplifies the hypervariable complementarity-determining region 3 (CDR3) regions of the TCR and employs high-throughput sequencing to characterize thousands of TCR CDR3 chains simultaneously.^34^ The technology can be applied to both cDNA and genomic DNA; when genomic DNA is used, the frequency of sequenced CDR3 chains is highly representative of the relative frequency of each T-cell containing CDR3 sequence in the biologic sample. Given the capacity of high-throughput sequencing, this assay is extremely sensitive and is applicable to a very low DNA yield. The assay demonstrates an outstanding specificity and sensitivity (~100-fold greater compared to other methods including flow cytometry). ImmunoSEQ is a highly accurate and standardized method for the assessment of TCRB diversity (*e.g.*, evenness of T-cell clones), clonality (abundance of specific clones), and T-cell abundance in general in normal or malignant tissues, and has been applied to understand and correlate the extent and diversity of T-cell infiltration postimmunotherapy with clinical outcome.^8,35–37^

Since the ultimate goal of T-cell-directed therapies is to facilitate recruitment and activation of tumor-specific T cells in the vicinity of tumor cells in target organs (*e.g.*, primary and metastatic tumor lesions, bone marrow) and biological fluids (*e.g.*, blood, ascites), local T-cell infiltration is a functional readout of T-cell reactivity as a result of therapy. Indeed, IHC analysis of CD3+ and CD8+ T cells pre- and on-treatment biopsies has demonstrated excellent clinical utility as pharmacodynamic biomarker in a number of trials investigating efficacy of genetically modified T cells and immunomodulatory agents.^8,30,38^

## Multiplex Pharmacodynamic Approaches to Monitor T-Cell Function and Bioactivity

Peripheral pharmacodynamic approaches have been applied to successfully monitor and provide mechanistic insights into T-cell efficacy and toxicity. Multiparametric flow cytometry enables a reasonably comprehensive phenotyping of specific immune cell subsets as well as their activation, proliferation, and differentiation status.^39–41^ Mass cytometry (cytometry by time-of-flight) is a variation of flow cytometry in which antibodies are labeled with heavy metal ion tags rather than fluorophores. With 135 detection channels and technical potential to measure about 400–500 molecules per cell, cytometry by time-of-flight represents a tool capable of revolutionizing immunophenotyping by increasing the number of antibodies that can be combined in one assay, while eliminating the issue of optical spillover (the presence of signals from fluorescent antibody staining in multiple detectors of a cytometer, resulting in a loss of resolution sensitivity).^41^ Once mass cytometry is standardized across various research laboratories, it would enable expansion of immunophenotyping capabilities by interrogating an extreme complexity and dynamics of immune cell subsets in the body.^42^

T-cell therapy and subsequent activation of T cells are frequently accompanied by a release of soluble immune factors (*e.g.*, cytokines, chemokines, extracellular domains of immune receptors) into the circulation, and quantification of these markers in peripheral blood might inform about the quality and potency of the T-cell response. This approach may also inform about a potential mechanistic link that might exist between T-cell activation and toxicity associated with specific T-cell therapies. Multiplexed microbead immunoassay platforms are commonly used to monitor cytokine levels in the circulation, and blood samples are analyzed at various time points in light of transient alterations displayed by various immune factors after infusion of adoptive transferred T cells or upon treatment with immunomodulatory agents.

Broad cytokine profiling of serum collected from the peripheral blood was essential to diagnose molecular mechanisms underlying a severe adverse event, cytokine release syndrome (CRS), in patients with B-cell malignancies who received anti-CD19 CAR-modified T cells;^43^ this agnostic profiling led to the observation of marked elevations in soluble interleukin-2 receptor α (sIL2Ra), interleukin-6 (IL-6), interferon (IFN-γ), and interleukin-6 (IL-6) associated with the CRS, and led to the development and use of IL-6 receptor (IL-6R) antibody (tocilizumab) to ameliorate the CRS-related toxicity, a strategy that has since been applied more broadly to ameliorate CAR T cell- and BiTE (blinatumomab)-induced CRS without compromising treatment efficacy..^26,29,44,45^ High levels of IFN-γ or sIL2Ra are indicative of T-cell activation and can potentially serve as pharmacodynamic markers for T-cell therapies. Patients with adoptively transferred CAR-modified T cells may also exhibit elevated levels of other markers of T-cell activation and inflammation including IL-2, IL-5, IL-8, IL-12, IL-17, IL-21, MCP-1 (CCL2), MIP-1α (CCL3), MIP-1β (CCL4), RANTES (CCL5), MIG (CXCL9), IP10 (CXCL10), fractalkine (CX3CL1), G-CSF, GM-CSF, Flt-3L, IL-1Rα, and/or TNFα.^24–26,31,46–48^ Furthermore, patients treated with blinatomumab exhibit comparable alterations in circulating cytokine levels^45,49^ demonstrating the utility of this platform to evaluate pharmacodynamic measures of bioactivity in the context of this treatment modality. Patients subjected to preinfusion lymphodepletion prior to CAR T-cell therapy also display elevated levels of IL-7 and IL-15, two γ-chain cytokines that exert homeostatic functions.^48^ IL-7 and IL-15 mediate antigen-independent memory T-cell self-renewal that may facilitate subsequent engraftment of adoptively transferred CAR T cells through stimulation of T-cell proliferation and differentiation while suppressing regulatory T-cell populations.^2^ Longitudinal quantification of circulating cytokine levels in peripheral blood by multiplexed microbead assays has also demonstrated clinical utility in trials with immune checkpoint inhibitors.^38,50^

The ability to effectively interrogate tumor biopsies to identify pharmacodynamic and mechanistic correlates with treatment efficacy has been greatly facilitated by recent technical advancements in multiparametric/multispectral IHC analysis which have demonstrated the potential to digitally quantify expression of multiple proteins at a single cell level.^8,51^ Once standardized and optimized, such approaches may significantly improve immonophenotyping (immunoscoring) of tumor samples and better understand, both qualitatively and quantitatively, tumor immune contexture—location, density, and functional orientation of infiltrating immune cells and their correlation with clinical outcomes.^52^

While IHC methods are commonly used to monitor immune cell infiltration in solid tumors, they require repeated tumor tissue biopsies that display striking heterogeneity and do not provide accurate information about temporal and spatial distribution of immune cells. Therefore, there is an urgent need to develop more effective techniques to monitor tumor immune cell infiltration *in vivo*. High-contrast immuno-positron emission tomography (immuno-PET) using radiolabeled minibody fragments (scFv-CH3) against CD8 represents a novel, noninvasive method for evaluation of CD8+ T-cell distribution and monitoring T-cell-dependent responses to immunotherapies *in vivo*.^53,54^ This platform has been evaluated in preclinical murine models and has the potential for translation into the clinical setting. However, it should be noted that while CD8-specific immuno-PET would be useful for monitoring the efficacy of BiTEs, immTACs and immune checkpoint blockade, tracking CAR T cells would also require another marker to differentiate engineered from endogenous, unmodified T cells.

Assessment of T-cell infiltration and distribution by IHC and molecular imaging can be complemented by high-content and/or high-throughput gene expression profiling to evaluate molecular and phenotypic changes in tumor tissue and peripheral blood cells after treatments using adoptive T-cell transfer, T-cell redirection, as well as administration of immunomodulatory agents. nCounter analysis system (Nanostring), a powerful digital detection hybridization-based technology capable of highly multiplexed (up to 800 targets), direct profiling of individual transcripts in a single reaction without amplification. This platform can accurately evaluate changes in expression of immune-related genes in peripheral and tumor-infiltrating T cells in patients who were treated with adoptively transferred TCR-engineered T cells or immunomodulatory agents.^55^ nCounter analysis is capable of monitoring T-cell expansion, activation, and/or exhaustion thus providing important molecular insights into gene expression signatures associated with immune response, candidate predictive biomarkers and potential treatment options that may help enhance and/or restore T-cell reactivity.

In addition to nCounter analysis system, other novel gene expression platforms (human whole transcriptome arrays, targeted RNA sequencing (RNAseq), microfluidics-based qPCR, Quantigene Plex assay) have demonstrate clinical utility as robust tools to monitor molecular and phenotypic traits in peripheral and tumor-infiltrating T cells.^37,38,50,56,57^

GeneChip human transcriptome array 2.0 (HTA 2.0, Affymetrix), a high-resolution microarray, has been successfully employed to detect unique gene expression changes in peripheral T cells upon treatment with PD-1 mAb, CTLA-4 mAb or PD-1/CTLA-4 mAb combination;^50^ while cell cycle/proliferation gene expression signature was associated with CTLA-4 mAb monotherapy and PD-1/CTLA-4 mAb combination therapy, treatment with PD-1 mAb resulted in the upregulation of genes implicated in effector T- and NK-cell function. Furthermore, each of these treatments were accompanied by unique changes in T-cell gene expression, with the combination PD-1/CTLA-4 mAb combination therapy resulting in the most extensive gene expression changes; 442, 26, and 36 differentially expressed genes were identified in PD-1/CTLA-4 mAb combination, CTLA-4 mAb and PD-1 mAb cohorts, respectively. Of note, IFN-γ seemed to be the only gene that displayed upregulation in all three cohorts.

With the advent of NGS, whole-exome and whole-transcriptome sequencing platforms are increasingly being used to understand the role of genomic and nongenomic alterations in tumors and their impact on anticancer therapies.^58^ Several lines of evidence indicate that NGS platforms complemented with computational epitope prediction, mass spectrometry, and/or the tandem minigene library approaches can be used to identify CT antigens ectopically expressed in tumors and novel tumor neoantigens recognized by both CD8+ and CD4+ T cells..^58–62^ NGS has also been applied to demonstrate the link between tumor mutational and neoantigen burden and molecular smoking signature and clinical efficacy of immune checkpoint inhibitors.^63–65^ Furthermore, there is emerging evidence that host microbiota is capable of influencing tumor response to immune checkpoint blockade.^66,67^ NGS-based platforms (miSeq, 16S rRNA sequencing) are rapidly becoming foundational tools capable of surveying the genomes of entire microbial communities including microorganisms not amenable to *ex-vivo* culture. These methods will enable further understanding of the impact that host microbiome makes on immune response and clinical efficacy of T-cell therapies.

As mentioned earlier, immunoSEQ is a highly sensitive and very specific method that can be used for the assessment of T-cell infiltration and for identification and monitoring specific T-cell clones in peripheral blood and tumor biopsies. This method is of particular importance in light of the recent findings suggesting that adoptive T-cell therapies (*e.g.*, mesothelin-specific CAR-modified T cells and NY-ESO-1-specific TCR-engineered T cells) are capable of epitope spreading.^30,31^ It is hypothesized that tumor lysis and inflammation induced by CAR T cells seem to result in the release of tumor antigens that are presented by dendritic cells to T cells resulting in the activation and expansion of endogenous T-cell clones. Epitope spreading thus represents an additional indirect mechanism of action of genetically engineered T cells. In addition, sequencing of immunoglobulin heavy-chain genes (IGH) is also frequently used to monitor minimal residual disease in patients with B-cell malignancies treated with adoptive transfer of CAR T cells.

## Platforms to Assess Target Expression and Identify Mechanisms of Acquired Resistance

While TCR and CAR T-cell therapies demonstrate remarkable overall response rates in cancer patients, tumor relapses represent a challenge. Clinical experience with CD19 CAR T-cell therapies suggests two modes of tumor recurrence: antigen-positive and antigen-negative.^43^ One mechanism for acquired resistance in patients whose tumor cells retain membranous target expression is inadequate engraftment and persistence of CAR-modified T cells or impaired reactivity of adoptively transferred T cells potentially caused exhaustion.

Single target therapy is capable of selecting for and/or inducing tumor escape subclones that lack target tumor antigen.^30^ Molecular mechanisms that underlie this phenomenon remain largely unknown although emerging data suggest that tumor escape in these cases may be driven by preexisting tumor cell clones that display certain genetic alterations and alternative splicing of T-cell target. More recently, one mechanism through which resistance to CART19 therapy is mediated in ALL has been shown to be the selection of rare CD19 splice variants which retain functional CD19 antigen but lack the epitope recognized by the CAR construct.^68^ These observations highlight the importance for monitoring antigen expression in target tissues during treatment using high sensitivity molecular strategies that can identify at the molecular level treatment-relevant molecular alterations. NGS-based platforms (*e.g.*, exome and RNA sequencing) have been instrumental to the dissection of molecular mechanisms of acquired resistance to targeted agents, and this approach should be actively applied to identify mechanisms of tumor recurrence in patients with antigen-negative disease that persists upon treatment with TCR- and CAR-engineered T cells.

In conclusion, broad immune and molecular profiling efforts that employed robust laboratory techniques have enabled identification of biomarkers that paved the way to further clinical development of T-cell therapies. Careful evaluation of their clinical and therapeutic relevance will be required to ensure that promising biomarkers are appropriately employed to maximize the benefit to risk ratio in cancer patients treated with T-cell-based therapies. Since it is unlikely that clinical efficacy in these cases will be associated with a single biomarker, a major challenge for the field will be to establish high-throughput, high-content infrastructure to support comprehensive correlative analyses and enable rigorous clinical qualification in the context of therapies for which they define clinical utility.

## Figures and Tables

**Figure 1:**
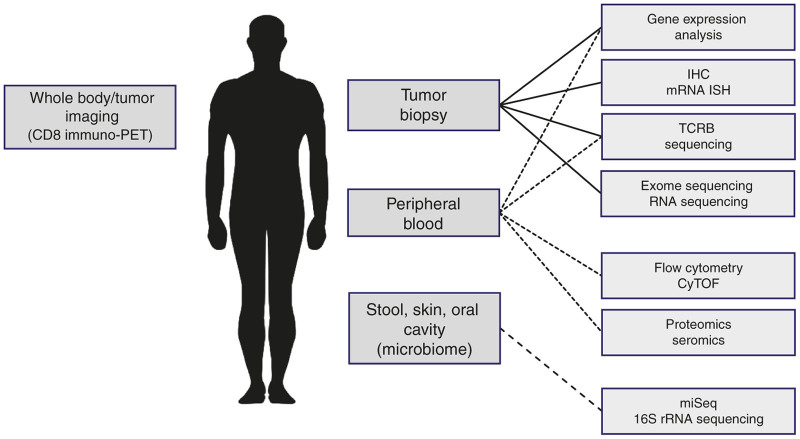
Scope and emerging platforms for translational research in immunooncology.
